# The financing need for expanding paid maternity leave to support breastfeeding in the informal sector in the Philippines

**DOI:** 10.1111/mcn.13098

**Published:** 2020-11-04

**Authors:** Valerie Gilbert Ulep, Paul Zambrano, Janice Datu‐Sanguyo, Mireya Vilar‐Compte, Graciela Ma Teruel Belismelis, Rafael Pérez‐Escamilla, Grace J. Carroll, Roger Mathisen

**Affiliations:** ^1^ Philippine Institute for Development Studies Quezon City Philippines; ^2^ Alive & Thrive Southeast Asia Hanoi Vietnam; ^3^ Research Institute for Equitable Development (EQUIDE) Mexico City Mexico; ^4^ Yale School of Public Health New Haven Connecticut USA

**Keywords:** breastfeeding, costing, informal sector, maternity leave, Philippines

## Abstract

In low‐ and middle‐income countries, almost three‐fourths of women in the labour force lack maternity protection. In the Philippines, current laws do not guarantee paid maternity leave to workers in the informal economy. A non‐contributory maternity cash transfer to informal sector workers could be used to promote social equity and economic productivity and could provide health benefits by helping mothers meet their breastfeeding goals. The objective of the study is to provide a realistic cost estimate and to assess the financial feasibility of implementing a publicly financed, non‐contributory maternity cash transfer programme to the informal sector in the Philippines. Using a costing framework developed in Mexico, the study estimated the annual cost of a maternity cash transfer programme. The methodology estimated the unit cost of the programme, the incremental coverage of maternity leave and expected number of enrollees. Different unit and incremental costs assumptions were used to provide a range of scenarios. Administrative costs for running the programme were included in the analysis. The annual financing need of implementing maternity cash transfer programme in the Philippines ranges from a minimum scenario of USD42 million (14‐week maternity cash transfer) to a more ideal scenario of USD309 million (26‐week maternity cash transfer). The latter is financially feasible as it is equivalent to less than 0.1% of the country's gross domestic product substantially lower than the share cost of not breastfeeding (0.7%). The annual cost of the programme is only 10% of the total cost of the largest conditional cash transfer programme.

**Key messages**

- In the Philippines, current laws do not guarantee paid maternity leave to workers in the informal economy. A publicly financed non‐contributory maternity cash transfer to informal sector workers could be considered.
- The annual financing need of implementing maternity cash transfer programme in the Philippines ranges from a minimum scenario of USD42 million to an ideal scenario of USD309 million.
- A publicly financed non‐contributory maternity cash transfer for informal sector seems financially feasible and a sound social investment in the Philippines.

## 1 INTRODUCTION

Exclusive breastfeeding is one of the most cost‐effective maternal and child health interventions (WHO and UNICEF, 2003). The World Health Organization (WHO) recommends exclusive breastfeeding for 6 months of life and continued breastfeeding with complementary foods until at least 24 months (Kramer & Kakuma, 2012). Despite its remarkable health benefits, the prevalence of exclusive breastfeeding remains low globally (Binns & Kung Lee, 2019; Development Initiatives, 2018). One of the major barriers to exclusive and continued breastfeeding is the lack of income protection for mothers, who then have no other choice but to rejoin the labour force soon after childbirth. Empirical studies have shown that the sooner mothers return to work, the shorter their breastfeeding duration (Bai, Fong, & Tarrant, 2015; Chang et al., 2019; ILO, 2019a; Nandi et al., 2016; Ogbuanu, Glover, Probst, Liu, & Hussey, 2011). Recent economic costing studies have also estimated the substantial annual economic burden resulting from not breastfeeding. In the Philippines, the cost of not breastfeeding is approximately 0.7% of gross domestic product (Walters, Phan, & Mathisen, 2019). The prevalence of exclusive breastfeeding in the Philippines is 27%, which is much lower than the global average (40%) (UNICEF, 2019), and off‐track from the Global Nutrition Target of at least 50% by 2025 (Development Initiatives, 2018).

Beyond its importance in establishing and maintaining breastfeeding, the provision of maternity leave is also essential to allow women to rest and physically and emotionally recover from giving birth (ILO, 2012).

Maternity leave duration in the Philippines was once the shortest in Southeast Asia at 60–78 days compared with the regional average of 3 months (ILO, 2019b). In 2015, only 55% of Filipino mothers received paid maternity leave as a form of income protection. Of those who received the leave, the average duration of leave was only 2 months (Food and Nutrition Research Insititute, 2016). About 40% of the 45 million in the labour force are women (Philippine Statistical Authority, 2019). Of the women in the labour force, around 39% are considered employed in the informal sector, and this estimate increases to 52% when short‐term and seasonal workers are included (Philippine Statistical Authority, 2017).

Across the working life cycle, labour force participation of women in the country has been consistently lower compared with those of men's, with the widest gap observed within the ages 20–39 years. This is the period when childbearing among women is most common and, in the Philippines, marriage and childbearing are associated with a significant decline in the female labour force participation. The provision of adequate maternity entitlements can make childbirth more compatible with economic activity and can facilitate the integration of women into the labour market (Cabegin & Gaddi, 2019). Maternity protection has a clear benefit to maternal and child health, and in the achievement of the country's Sustainable Development Goals (SDG) and its targets on maternal and child health, and nutrition, and gender equality.

In 2019, the Philippine Congress enacted a landmark law (Expanded Maternity Leave Act of, 2019) that extended paid maternity leave to 15 weeks, in alignment with the International Labor Organization convention of at least 14 weeks (ILO, 2019a). Despite this positive development, full and equitable implementation of the law will be challenging. The Expanded Maternity Leave Act of 2019 only guarantees maternity leave to mothers working in the formal sector. Workers in the informal sector, while explicitly covered by the law, could only be eligible if they are members of the Social Security System, the state‐run social insurance programme for private and informal sector workers. However, a large number of women in the informal sector are not members of the social insurance programme (National Economic Development Authority, 2017). In 2015, only 12% of self‐employed (i.e., in Philippines considered to be employed by the informal sector) mothers received maternity leave. Also, a large percentage of mothers who reported working in the government and private sectors did not receive paid maternity benefits (25% and 45%, respectively) (Food and Nutrition Research Insititute, 2016). Short‐term and seasonal workers typically have less access to social protection including maternity leave (National Economic Development Authority, 2017).

Given the substantial number of women working in the informal sector in the Philippines, a large number of children and mothers continue to be vulnerable to health risks due to lack of protection resulting in not breastfeeding according to recommendations. To address this gap, the government could extend a publicly financed non‐contributory maternity cash transfer programme to the informal sector. As evidence suggests, providing financial incentives to mothers, particularly low‐income earners, will improve breastfeeding practice (Clay, Strong, & Thomas, 2018; Mirkovic, Perrine, & Scanlon, 2016). However, cash transfer programmes are typically perceived as costly, which could lead to political resistance. Following an innovative costing framework, this study provides a realistic estimate on the cost requirements of a publicly financed non‐contributory maternity cash transfer programme in the Philippines and presents these costs in relation to costs of not breastfeeding and its consequences. These findings provide critical inputs in assessing the financial feasibility of extending income protection to mothers in the informal sector.

## 2 METHODOLOGY

To estimate the annual financing need of a maternity cash transfer programme for the informal sector workers, we adapted the formula developed by Villar‐Compte et al. (2019):

$${\mathrm{MCT}}_{y}={\mathrm{UC}}_{\mathrm{CT}}\;*{\mathrm{IC}}_{y}*\left(\alpha *{\mathrm{Pop}}_{y}\right)+{\text{AdmC}}_{y},$$

where

*MCT*_*y*_ is the cost of maternity cash transfer for the informal sector on a given year *y*;

*UC*_*CT*_ is the unit cost (UC) of weekly maternity cash transfer (per week/per mother);

*IC*_*y*_ is the incremental coverage of cash transfer (number of weeks) for year *y*;

*α* is the probability of having a baby and working in the informal sector;

*Pop*_*y*_ is the number of women of reproductive age in the country for year *y*;

*AdmC*_*y*_ is the total administrative cost of the programme for year *y*.

The formula provides a realistic estimate of the total cost of maternity cash transfer programme because it weights the population of women currently working in the informal sector considering the mothers' education, age, location and marital status. The costing modelling approach followed included six clearly delineated steps. To estimate the parameters required from each step, we used employment and fertility survey data, census data and official government data reporting on minimum wage, average daily wage and poverty line threshold. To ensure that our estimates from surveys reflected population parameters, we applied survey weights. Our cost estimates were converted to USD and reported in 2018 prices.

In Step 1, we estimated the probability of having a child in the prior year among women of reproductive age using the 2017 Philippine National Demographic and Health Survey (NDHS) (Philippine Statiscal Authority and ICF, 2018). The analysis was categorized according to maternal age (15–24, 25–29, 30–34, 35–39 or 40–49 years), marital status (single, married, widowed or divorced/annulled/separated), type of residence (urban or rural) and educational attainment (no education/preparatory, primary, secondary or tertiary/higher). Given these characteristics, we generated 160 unique combinations of groups according to their demographic characteristics (e.g., women aged 18–24 years old, single, living in rural area and high school graduate). For each combination, we estimated the probability of having a child in the prior year. Mothers with infant deaths were excluded from the analysis.

In Step 2, we estimated the probability of being employed in the informal sector among women of reproductive age. The 2017 NDHS does not contain detailed information on employment; hence, we used the 2017 Labor Force Survey (Philippine Statistical Authority, 2017) to determine the estimated proportion of women of reproductive age participating in the labour force and the proportion working in the informal sector. The analysis was categorized according to the same 160 unique subgroups as explained above based on maternal age, marital status, type of residence and educational attainment. We defined informality based on what is contextually relevant in the Philippines. Social protection schemes in the Philippines define formal workers as those who are working for government and private establishments or corporations. The government mandates public and private establishments/corporations to provide maternity benefits to employees. Operationally, we classified women as being employed in the formal sector if they worked in a private or government establishment, family‐owned farm with pay or private household (including domestic work). In our analyses, we classified self‐employed or workers in family‐owned farm without pay as informal sector workers. Additionally, we expanded the definition of informal sector to include mothers with short‐term and seasonal employment—groups of female workers who are unreached by existing maternity protection benefits and can potentially benefit from a maternity cash transfer. We argue that this definition is more inclusive from a social justice perspective. Mothers who were unemployed were excluded in the analysis.

Using the probabilities obtained in Steps 1 and 2, we obtained an estimate of alpha, which is the probability of a woman of reproductive age, working in the informal sector, of having a child in the previous year for each of the 160 subgroups.

In Step 3, we estimated the weighted population of women using the, α * Pop_*y*_. The value is a realistic estimation of the women employed in the informal sector who could claim maternity leave in a given year. We used the 2017 projected population of women of reproductive age from the Philippine Statistical Authority.

In Step 4, we multiplied the weighted population by the UC of the maternity cash transfer. We used three levels of UCs based on minimum wage, average daily salary and poverty threshold. To derive the weekly cost, we multiplied the reported minimum wage and average daily salary by five and divided the reported biannual poverty threshold by 24. The minimum wage is mandated by the Labor Code of the Philippines. We used the reported minimum wage and average daily salary from the 2018 Department of Labor and Employment, while we used the official poverty threshold from Philippine Statistical Authority, respectively (Philippine Statistical Authority, 2019). The Statistical Authority defines poverty threshold as the minimum expenditure required for an individual to meet the basic food and non‐food requirements.

In Step 5, we added the incremental coverage of the maternity cash transfer programme (i.e., number of weeks covered). We used the following incremental coverages: (1) 14 weeks, which is the minimum as mandated by ILO convention; (2) 15 weeks, which is the length of maternity leave as mandated by Expanded Maternity Leave Act of, 2019; (3) 18 weeks, which is the more optimal duration as stipulated in ILO Recommendation 191 (ILO, 2019a); (4) 26 weeks after delivery, which is consistent with the recommendations of the WHO for exclusive breastfeeding. We obtained a range of yearly maternity leave cash transfer costs for 12 scenarios based on the combination of three levels of UCs and four levels of incremental costs.

In Step 6, we added the administrative cost of implementing the maternity cash transfer programme. This cost is a proxy and is based on the audited financial statements of two major social protection programmes in the country: Social Security System, the mandatory pension programme for private sector employees; and Philippine Health Insurance Corporation, the primary national health insurance programme in the country. Both programmes are well‐established government‐controlled corporations' and PhilHealth cover 16 and 50 million members (excluding beneficiaries of members) and, in 2017, spent 15% and 17% of the corporations' total expenses on administrative cost, respectively. The average share of administrative cost (16. 3%) was applied in our estimates (Philippine Health Insurance Corporation, 2017; Social Security System, 2017). We used the lowest administrative cost across all incremental coverage scenarios (i.e., 14 weeks). We assumed that the administrative cost across scenarios are the same. This is a realistic assumption because an increase in the incremental coverage does not necessarily lead to additional administrative cost.

## 3 RESULTS

Table 1 shows the probability of women of reproductive age of having a baby in the prior year obtained from the 2017 NDHS and the percentage of informal workers among economically active women of reproductive age (i.e., employed) from 2017 Labor Force Survey. Women from younger age group, primary/secondary education graduate, who are married, and from rural area were more likely to have birth in the prior year, whereas women with no education, married and from rural area were more likely to be working in the informal sector. Table 1 only shows the systematic differences in fertility and labour force and the importance of considering socio‐demographic characteristics in the model. Based on our estimates, there were 24,471,807 women of reproductive age (15–49 years old), but only 11,060,355 of these women were in the labour force. Out of these, 238,924 were in the informal sector and had infants in the previous year.

**TABLE 1:** Characteristics of women of reproductive age and in the labour force

Demographic variable	Category	With birth in last year (%)			Informality (%)		
		%	95% LL	95% UL	%	95% LL	95% UL
Age	15–24	6.2	5.7	6.6	18.9	17.7	20.0
	25–29	7.0	6.2	7.7	25.7	24.2	27.2
	30–34	4.0	3.3	4.6	37.3	35.6	39.9
	35–39	2.0	1.6	2.5	42.4	40.8	44.7
	40–44	0.3	0.2	0.4	48.8	47.7	49.6
	45–49	0.2	0.1	0.3	49.7	48.1	51.3
Educational attainment	No education, preschool	2.9	1.3	4.5	63.2	61.1	65.4
	Primary	3.9	3.3	4.5	52.3	50.9	53.8
	Secondary	4.4	4.1	4.8	37.6	36.5	38.7
	Higher	2.9	3.4	4.2	37.9	35.9	40.0
Marital status	Never married	1.0	0.8	1.1	16.4	15.5	17.3
	Married	6.1	5.7	6.5	46.4	45.6	47.3
	Widowed	0.8	−0.1	1.6	38.5	34.7	42.2
	Divorced/separated	3.4	2.3	4.5	26.4	23.2	29.5
Type of residence	Urban	3.8	3.4	4.2	26.4	25.6	27.2
	Rural	4.4	4.1	4.7	46.3	45.4	47.7

Table 2 shows the maternity leave cash transfer costs using the formula*: MCT*_*y*_ *= UC*_*CT*_ * IC_*y*_ * (a * Pop_*y*_) with different scenarios of incremental coverage and UC of maternity cash transfer. The table also includes marginal cost per week for each type of cash transfer. Depending on the welfare measure, the marginal cost per week ranges from USD2.5 million to USD10.8 million.

**TABLE 2:** Maternity leave cash transfer (MCT) costs per extent in weeks (in USD) using exhaustive definition of informal sector

Unit cost	Incremental coverage				
	Cost per week	14 weeks	15 weeks	18 weeks	26 weeks
Poverty line	2,483,994	34,775,922	37,259,917	44,711,900	64,583,856
Minimum wage	10,760,816	150,651,421	161,412,234	193,694,684	279,781,210
Basic pay	9,840,458	137,766,416	147,606,874	177,128,248	255,851,915

*Note*. Exchange rate: 50. 40USD = 1PhP (World Development Indicators) in 2018 prices.

Table 3 shows the total amount of maternity benefits including the administrative cost in running the programme using the formula: *MCT*_*y*_ *= UC*_*CT*_ * IC_*y*_ * (a * Pop_*y*_) *+ AdmC*_*y*_.

**TABLE 3:** Total cost of the maternity leave cash transfer per year (in USD) using exhaustive definition of informal sector

Variable	Administrative cost^a^	Cash transfer^a^	Total cost^a^	Cost per woman^b^
Poverty line				
14 weeks	6,747,567	34,775,922	41,523,490	174
15 weeks	6,747,567	37,259,917	44,007,484	184
18 weeks	6,747,567	44,711,900	51,459,467	215
26 weeks	6,747,567	64,583,856	71,331,423	299
Minimum wage				
14 weeks	29,230,873	150,651,421	179,882,294	753
15 weeks	29,230,873	161,412,234	190,643,107	798
18 weeks	29,230,873	193,694,684	222,925,557	933
26 weeks	29,230,873	279,781,210	309,012,082	1,293
Average daily salary				
14 weeks	26,730,797	137,766,416	164,497,214	688
15 weeks	26,730,797	147,606,874	174,337,671	730
18 weeks	26,730,797	177,128,248	203,859,045	853
26 weeks	26,730,797	255,851,915	282,582,713	1,183

*Note*. Poverty line: PhP524 per week; minimum wage: PhP2,270 per week; average daily salary: 2,076 per week.

^a^ Exchange rate: 50.40USD = 1PhP (World Development Indicators) in 2018 prices.

^b^ A total of 238,924 were in the informal sector and had infants in the previous year.

We conducted a sensitivity analysis of total cost of the maternity leave cash transfer per year (Table 4). This sensitivity analysis examined the changes in our estimates when the following scenarios are applied in the model: (1) a less exhaustive definition of informality and (2) assume that 12% informal sector workers have maternity protection. In our analysis, we used an exhaustive definition of informality. We categorized short‐term and seasonal workers even among traditionally defined as formal sector workers (e.g., workers in government and private corporations) as informal. If we remove this restriction, the estimated 14‐week maternity cash transfer using minimum wage as UC is down to USD136 million from USD180 million.

**TABLE 4:** Sensitivity analysis of total cost of the maternity leave cash transfer per year (in USD)

Variable	Exhaustive definition of informality (A)^a^	Less exhaustive definition of informality (B)^a^	Informal sector with maternity protection (C)^a^
Poverty line			
14 weeks	41,523,490	31,673,588	40,715,094
15 weeks	44,007,484	33,454,018	43,141,345
18 weeks	51,459,467	38,795,308	50,420,101
26 weeks	71,331,423	53,038,749	69,830,116
Minimum wage			
14 weeks	179,882,294	136,260,544	175,083,801
15 weeks	190,643,107	143,905,520	185,501,868
18 weeks	222,925,557	166,840,450	216,756,067
26 weeks	309,012,082	228,000,261	300,100,597
Average daily salary			
14 weeks	164,497,214	125,476,372	163,794,785
15 weeks	174,337,671	132,529,627	173,406,493
18 weeks	203,859,045	153,689,393	202,241,617
26 weeks	282,582,713	210,115,435	279,135,280

*Note*. A: Exhaustive definition: employed mothers who are as self‐employed or employed in family‐owned farm or private household farm. Regardless of employment type, if they work is further as short‐term or seasonal, they are considered as informal. B: Less exhaustive definition: same as (A), but seasonality or duration of work not considered. C: Informal sector: Same as A, but 12% of informal sector are excluded. A study show that 12% informal sector availed maternity leave benefits.

^a^ Exchange rate: 50.40USD = 1PhP (World Development Indicators) in 2018 prices.

Based on the reported estimates of the Food and Nutrition Research Institute, around 12% of mothers in the informal sector received maternity benefits (Food and Nutrition Research Insititute, 2016). Applying this weight to the estimated number of enrollees yields to negligible difference (A vs. C). As expected, using a less exhaustive definition yielded to lower estimated cost (A vs. B). The estimated 14‐week maternity cash transfer using minimum wage as UC went down from USD180 to USD175 million (see Table 4).

## 4 DISCUSSION

Our study estimated the annual cost requirement of a maternity cash transfer programme targeting female informal sector workers considering the number of women the programme would cover in a given year. At the minimum, the Philippine government needs to spend about USD42 million (or USD174 per mother) for a 14‐week maternity leave using the poverty line as UC. The cost increases to USD 180 million (or USD 753 per mother) for a 14‐week equivalent maternity leave using minimum wage as UC. To contextualize, the estimated total cost of USD 180 million for a 14‐week maternity cash transfer is less than 0.06% of the country's gross domestic product, 9% of the total budget of the Department of Health or 10% of the total budget of the largest conditional cash transfer programme for the poor, the *Pantawid Pamilyang Pilipino Program* (Department of Budget and Management, 2019). The share is lower (two percentage point) if the poverty threshold is used as UC, suggesting that providing informal workers with a maternity cash transfer benefit could be affordable. In the medium to long‐term, the total cost of maternity cash transfer programme expected to dramatically decrease as more women are exiting the informal economy. From 2010 to 2018, the share of informal workers to total labour force has declined 3% annually (World Bank, 2020). These investments should also be put in the context of the country's development agenda and its commitments to broader human rights instruments. Income security is critical to enable mothers to recover before and after childbirth and support exclusive and continued breastfeeding, thereby preventing health risks for women and their children (ILO, 2016) and to ensure that both mother and child benefit from the short and long‐term outcomes associated with breastfeeding. Because breastfeeding is a major driver of many SDGs, improving maternity protection for informal sector workers complements the strategy of the Philippines to achieve its targets on maternal and child health, and nutrition. Breastfeeding rates remain suboptimal, and under‐five mortality remains high in the Philippines relative to other countries with similar economic development. Our estimated cost of the maternity cash transfer is outweighed by the potential economic benefit of improved breastfeeding rates at population level. For example, studies have shown an inverse relationship between maternity benefits and under‐five mortality a (Nandi et al., 2016). Indeed, according to Walters et al. (2019) estimates, our estimated cost of a 14‐week maternity leave is only 5% of the cost of not breastfeeding in the Philippines.

The right to maternity protection is enshrined in different Philippine laws. The country is also signatory of international human right treaties such as the Universal Declaration of Human Rights (UDHR), the International Covenant on Economic, Social and Cultural Rights and the Convention on the Elimination of All Forms of Discrimination against Women (CEDAW). All these laws and treaties provide legal and political justifications for expanding maternity protection for all mothers regardless of employment status. For example, the International Covenant on Economic, Social and Cultural Rights recognizes maternity protection, including non‐discrimination, maternity leave, and childcare as essential rights. Improving maternity protection coverage for informal sector workers will represent a significant step to meeting the country's obligations to these human rights instruments.

The potential role of adequate maternity entitlements in facilitating the integration of women into the labour market cannot be understated, especially considering that in the Philippines, female labour force participation as of 2017 is relatively low (45.2%) and is significantly lower than labour force participation of males (76.1%) (The Association of Southeast Asian Nations, 2018). Equitable maternity protection entitlement thus also contributes the country's achievement of SDG targets on gender equality.

Women in the informal sector play an important role not only to the well‐being of their households but also to the growth of the national economy (Chen, 2001). Despite their economic contributions, women in the informal sector face structural constraints, including limited income and social protection during pregnancy and after childbirth. A maternity leave benefit is an important component of the social protection system alongside with other nonincome‐based benefits.

In recent years, there is a growing recognition on the importance of early child nutrition in the Philippines. In 2019, the Congress enacted a law, which scales up nutrition programmes through a strengthened strategy for maternal, neonatal and child health nutrition in the first 1,000 days of life. Innovative approaches such as maternity cash transfers will enable equitable implementation of such maternity protection policies, reaching what has been an underserved but substantial proportion of female workers.

It is important to acknowledge that even though cash transfer programmes have the reach in many countries to deliver the proposed maternity benefit among women working it the informal economy, and they have been proven to benefit the health, nutrition and development of young children (Segura‐Perez & Perez‐Escamilla, 2006), it is possible that they can have unintended effects in recipient families (Fernald, Gertler, & Hou, 2008; Nandi & Laxminarayan, 2016) For this reason, it is important to tailor the design of the delivery of the proposed benefit to the different social, economic and cultural contexts where the cash transfer programmes operate.

Our study has limitations. First, our employment data do not capture membership in the social protection scheme, which prevented us from excluding ineligible women in our estimation. The inclusion of self‐employed women with some form of maternity protection possibly led to an overestimated cost. However, we attempt to address this in our sensitivity analysis, we adjusted our estimates by excluding mothers with maternity protection among informal sector workers using a factor from a published report. Second, our study used a proxy for the estimated administrative cost based on existing social protection schemes with published financial information. The share of administrative cost to total expenditure we used in the analysis appears to be high compared with other social protection schemes in low‐ and middle‐income countries (Villar‐Compte et al., 2019). However, we chose the share of administrative cost from existing social protection schemes because they are more contextually relevant. Further studies are needed to do a more in‐depth estimation of administrative costs in the Philippines, which can then be easily modelled with the flexible algorithm that we have developed.

For future research, decision makers may benefit from costing estimates disaggregated by socio‐demographic status (e.g., mothers who are poor and young mothers in the informal sector). This may provide information especially for more a targeted and progressive income transfer programme.

## 5 CONCLUSION

Our study proposes and costs a publicly financed non‐contributory maternity protection programme for informally employed women. Although the political and institutional feasibility of such programme needs further assessment, its cost seems affordable especially in light of the economic gains from positive health and nonhealth outcomes associated with improved breastfeeding rates and female labour force participation. The Philippines had a long history of implementing and managing income protection schemes. Also, there are already existing social protection schemes that can be used to properly tailor a maternity cash transfer programme in the Philippine context.

## CONFLICTS OF INTEREST

The authors declare that they have no conflicts of interest.

## CONTRIBUTIONS

The authors' responsibilities were as follows: VGU analysed and wrote the first draft. VGU, PZ and JDS interpreted the data and contributed to the revisions. PZ, RM and JDS developed the overall concept of the project. MCV, GC, GTB and RPE developed the methodology and contributed to the revisions. RM and RPE provided critical intellectual feedback to help revise the manuscript and contributed to the revisions. All authors have read and approved the final manuscript.
